# Activation of 2‐oxoglutarate receptor 1 (OXGR1) by α‐ketoglutarate (αKG) does not detectably stimulate Pendrin‐mediated anion exchange in *Xenopus* oocytes

**DOI:** 10.14814/phy2.15362

**Published:** 2022-07-18

**Authors:** John F. Heneghan, Amar J. Majmundar, Alicia Rivera, Jay G. Wohlgemuth, Jeffrey S. Dlott, L. Michael Snyder, Friedhelm Hildebrandt, Seth L. Alper

**Affiliations:** ^1^ Division of Nephrology Beth Israel Deaconess Medical Center Boston Massachusetts USA; ^2^ Department of Medicine Harvard Medical School Boston Massachusetts USA; ^3^ Division of Nephrology Boston Children's Hospital Boston Massachusetts USA; ^4^ Department of Pediatrics Harvard Medical School Boston Massachusetts USA; ^5^ Quest Diagnostics Secaucus New Jersey USA; ^6^ Department of Genetics Harvard Medical School Boston Massachusetts USA

**Keywords:** cortical collecting duct, GPR99, kidney, type B intercalated cell

## Abstract

SLC26A4/Pendrin is the major electroneutral Cl^−^/HCO_3_^−^ exchanger of the apical membrane of the Type B intercalated cell (IC) of the connecting segment (CNT) and cortical collecting duct (CCD). Pendrin mediates both base secretion in response to systemic base load and Cl^−^ reabsorption in response to systemic volume depletion, manifested as decreased nephron salt and water delivery to the distal nephron. Pendrin‐mediated Cl^−^/HCO_3_^−^ exchange in the apical membrane is upregulated through stimulation of the β‐IC apical membrane G protein‐coupled receptor, 2‐oxoglutarate receptor 1 (OXGR1/GPR99), by its ligand α‐ketoglutarate (αKG). αKG is both filtered by the glomerulus and lumenally secreted by proximal tubule apical membrane organic anion transporters (OATs). OXGR1‐mediated regulation of Pendrin by αKG has been documented in transgenic mice and in isolated perfused CCD. However, aspects of the OXGR1 signaling pathway have remained little investigated since its original discovery in lymphocytes. Moreover, no ex vivo cellular system has been reported in which to study the OXGR1 signaling pathway of Type B‐IC, a cell type refractory to survival in culture in its differentiated state. As *Xenopus* oocytes express robust heterologous Pendrin activity, we investigated OXGR1 regulation of Pendrin in oocytes. Despite functional expression of OXGR1 in oocytes, co‐expression of Pendrin and OXGR1 failed to exhibit αKG‐sensitive stimulation of Pendrin‐mediated Cl^−^/anion exchange under a wide range of conditions. We conclude that *Xenopus* oocytes lack one or more essential molecular components or physical conditions required for OXGR1 to regulate Pendrin activity.

## Graphical abstract

We tested the ability of α‐ketoglutarate to activate Pendrin‐mediated anion exchange in Xenopus oocytes coexpressing OXGR1 and Pendrin. We found that oocytes did not reproduce a‐ketoglutarate's stimulatory activity on pendrin previously reported in mice and isolated perfused mouse collecting duct.

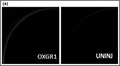

## 1 INTRODUCTION

SLC26A4/Pendrin is the electroneutral Cl^−^/HCO_3_^−^ exchanger of the lumenal membrane of Type B intercalated cell of the renal collecting duct (Royaux et al., 2001; Wall et al., 2020). Pendrin secretes excess bicarbonate into the lumen in response to systemic alkaline load, and reabsorbs Cl^−^ under conditions of systemic salt load (Wall, 2022). In perfused mouse collecting duct, accompanying Na^+^ can be transported by electroneutral SLC4A8/NDCBE (Leviel et al., 2010). Studies in perfused rat collecting duct show Na^+^ reabsorption via ENaC upregulation and, perhaps, also through paracellular pathways (Pech et al., 2015).

Although Pendrin knockout mice have congenital deafness accompanied by enlargement of the vestibular aqueduct (Alper & Sharma, 2013; Ito et al., 2013; Roesch et al., 2021), they are grossly normal in appearance and in baseline renal phenotype, and do not develop a normal hypertensive response to high‐dose aldosterone (Verlander et al., 2003). However, pendrin—NCC double knockout mice are highly susceptible to volume depletion (Soleimani et al., 2012). Patients with Pendred Syndrome caused by recessive hypomorphic pendrin mutations, are normotensive but may rarely be subject to life‐threatening hypovolemia in the setting of viral infections with GI fluid loss (Kandasamy et al., 2011). Based on pendrin's contribution to as much as 50% of NaCl reabsorption in mouse connecting segment and/or cortical collecting duct (Leviel et al., 2010), pendrin is a strong candidate therapeutic target for the development of new diuretics (Cil et al., 2016) and other clinical applications (Verkman & Galietta, 2021).

The α‐ketoglutarate (αKG) receptor OXGR1/GPR99 was first identified through its homology with the succinate receptor GPR91 (He et al., 2004). OXGR1 was shown to be expressed at the highest levels in kidney, where it localized to the distal nephron. It was postulated to signal via Gαq pathways on the basis of HEK‐293 cell overexpression studies, which revealed αKG‐induced, pertussis toxin (PTX)‐insensitive elevations in concentrations of intracellular Ca^2+^ ([Ca^2+^_i_]) and of phosphoinositols, without elevation of cAMP levels (He et al., 2004). More recently, OXGR1 was shown to be localized to Type B and non‐A non‐B intercalated cells of the cortical collecting duct (CCD) (Tokonami et al., 2013), with enrichment in the luminal membrane (Diehl et al., 2016; Grimm et al., 2015). OXGR1 is believed to bind αKG secreted across the lumenal membrane of proximal tubular epithelial cells (Tokonami et al., 2013) by OAT10/SLC22A13 (Grimm et al., 2015) and perhaps by other organic anion transporters. In isolated perfused mouse CCD, aKG‐stimulated OXGR1 increases Pendrin‐mediated Cl^−^ reabsorption across the cortical collecting duct via a Ca^2+^‐dependent, PKCα‐ or PKCδ‐dependent pathway or via a PKC‐independent, SPAK‐mediated pathway to maintain systemic volume or to contribute to pathological systemic salt and volume overload (Lazo‐Fernandez et al., 2018).

Although the ability of OXGR1 to upregulate Pendrin activity in intact mice and in intact perfused cortical collecting ducts of mouse has been established (Lazo‐Fernandez et al., 2018; Tokonami et al., 2013), and some elements of the mechanism of stimulation have been revealed (Lazo‐Fernandez et al., 2018), the ability of OXGR1 to activate Pendrin in a more easily manipulated heterologous expression system has not been reported. As we have previously studied Pendrin transport function in *Xenopus* oocytes (Reimold et al., 2011), we tested the *Xenopus* oocyte as a potentially convenient experimental system in which to study the ability of co‐expressed OXGR1 to upregulate Pendrin‐mediated Cl^−^/anion exchange.

## 2 METHODS

### 2.1 Materials

cDNA encoding human OXGR1 was obtained from AddGene (Watertown, MA) and subcloned into oocyte expression plasmid pGEMHE. cDNA encoding human SLC26A4/Pendrin in oocyte expression plasmid pXT7 was previously described (Reimold et al., 2011). Plasmid cDNAs encoding Gα protein subunits were obtained from the cDNA Resource Center. Plasmids encoding C‐terminally MYC‐DDK‐tagged human SLC4A9 (Origene RC212283) and MYC‐DDK‐tagged mouse SLC4A9 in pCMV6‐Entry (Origene MR21228) (Pena‐Munzenmayer et al., 2016) were kind gifts of Prof. Marcelo Catalan (Univ. Arturo Prat). Oocyte expression plasmid pIN‐TRPV5, encoding human TRPV5 (Zheng et al., 2018), was the kind gift of Ruiqi Cai and Xing‐Zhen Chen (Univ. Alberta) and Ji‐Bin Peng (Univ. Alabama). Na^36^Cl was from PerkinElmer. Restriction enzymes and T4 DNA ligase were from New England Biolabs. EXPAND High‐fidelity PCR System was from Roche. Other reagents were from Sigma‐Aldrich or Fluka.

### 2.2 Solutions

MBS consisted of (in mM) 88 NaCl, 1 KCl, 2.4 NaHCO_3_, 0.82 MgSO_4_, 0.33 Ca(NO_3_)_2_, 0.41 CaCl_2_, and 10 HEPES (pH 7.4). ND‐96 consisted of (in mM) 96 NaCl, 2 KCl, 1.8 CaCl_2_, 1 MgCl_2_, and 5 Na HEPES (pH 7.4). NMDG‐97 consisted of (in mM) 97.3 N‐methyl‐D‐glucamine (NMDG) chloride, 2.0 KCl, 1.2 CaCl_2_, 1 MgCl_2_, and five HEPES free acid (final pH 7.4). Cl^−^ substitution was achieved by equimolar replacement of NaCl with Na cyclamate. Cl^−^ salts of K^+^, Ca^2+^, and Mg^2+^ were substituted as needed with the corresponding equimolar gluconate salts.

### 2.3 cRNA synthesis and expression in xenopus oocytes

Capped cRNA was transcribed at 37°C from linearized cDNA template encoding human OXGR1 or human SLC26A4/Pendrin using the Megascript T7 kit (Life Technologies), purified with the RNeasy mini‐kit (Qiagen), and quantitated by Nanodrop spectrometer (ThermoFisher). The same procedure was used for linearized plasmids encoding SLC4A9 and TRPV5. RNA integrity was verified by formaldehyde gel electrophoresis. Mature female *Xenopus laevis* (Dept. Systems Biology, Harvard Med. School or NASCO) were subjected to partial ovariectomy under hypothermic tricaine anesthesia following protocols approved by the Institutional Animal Care and Use Committee of Beth Israel Deaconess Medical Center. Stage VI oocytes were prepared by overnight incubation of ovarian fragments in MBS with 2 mg/mL collagenase B (Alfa Aesar), followed by a 20 min rinse in Ca^2+^‐free MBS, with subsequent manual selection and defolliculation as needed. Oocytes were injected with cRNA on the day of isolation and maintained at 17.5°C in MBS supplemented with gentamicin (10 μg/mL) for 72 h.

### 2.4 Confocal immunofluorescence microscopy

Oocytes were injected with 50 ng human MYC‐tagged OXGR1 cRNA. cRNA‐injected oocytes and uninjected oocytes were incubated 72 h at 17.5°C in MBS containing gentamicin (10 μg/mL). Ten oocytes in each experimental group were fixed in 3% paraformaldehyde (PFA) in phosphate‐buffered saline (PBS) for 30 min at room temperature, washed three times with PBST, permeablized with 1% SDS in PBS supplemented with 0.02% Na azide (PBS‐azide) for 1 to 2 minutes, and washed again three times with PBST.

Fixed, permeabilized oocytes were incubated overnight at 4°C in 0.5 ml PBST containing affinity‐purified polyclonal rabbit anti‐Myc Ig (Cell Signaling #71D10) at 1:1000 dilution, rinsed in PBST, followed by Cy3‐conjugated donkey‐anti‐rabbit secondary Ig (Cell Signaling) for 90 min at 20°C. Stained oocytes were washed 3x in PBST, 2x in PBS‐azide, and post‐fixed in PFA for 10 min. Fixed oocytes were washed 2x in PBST, extensively washed with PBS‐azide, and stored in PBS‐azide at 4°C until imaged. Cy3‐labeled oocytes aligned in uniform orientation along a plexiglass groove were sequentially imaged through the 10x objective of a Zeiss LSM510 laser scanning confocal microscope, using the 543 nm laser line at 512 × 512 resolution at a uniform setting of 80% laser intensity, pinhole 54 (1.0 Airy units), detector gain 650, Amp gain 1, zero amp offset.

Polypeptide abundance at or near the oocyte surface was estimated by quantitation of specific fluorescence intensity (FI) at the circumference of one quadrant of an equatorial focal plane image of the oocyte (Image J v. 1.38, National Institutes of Health). Background correction was performed by subtraction from the FI of each cRNA‐injected oocyte of the mean FI value of a comparable circumference area from an equatorial plane quadrant of water‐injected oocytes.

### 2.5 Oocyte lysate immunoblots

Twenty oocytes injected with OXGR1 cRNA (50 ng) were placed in 1.5 ml MBS and incubated 72 h at 17.5°C. MBS was then aspirated and replaced with RIPA buffer containing Complete Protease inhibitor (6 μl per oocyte), vortexed vigorously and immediately frozen at −80°C for ≥2 h. The subsequently thawed mixture was again vortexed, then centrifuged 20–30 min at 4°C at maximal microfuge speed. The opalescent infranatant separating pellet and the foamy supernatant was withdrawn and subjected to two more cycles of vortexing, 4°C centrifugation, and infranatant harvest. The final, clarified protein extracts were assayed for protein content by the BCA method and stored at −80°C until use. 20 μg of extract protein was brought to 10 μl volume with RIPA buffer containing protease inhibitors, and 4 μl SDS load buffer containing β‐mercaptoethanol was added. The sample mixture was incubated at room temperature for 30 min, then loaded on an 8–16% polyacrylamide gradient tris‐glycine gel (BIO‐RAD) and subjected to SDS‐PAGE. Protein was transferred to PVDF membrane (BioRad TurboBlot), washed in TBST, and blocked 1 h with TBST plus 5% powdered milk. The blocked membrane was washed with TBST and incubated overnight at 4°C with affinity‐purified rabbit polyclonal anti‐Myc Ig (Cell Signaling #71D10) diluted 1:500 in TBST/5% BSA, then further washed and incubated 1 h with horseradish peroxidase‐coupled goat anti‐rabbit Ig (Thermo Scientific #31460) diluted 1:8000 in TBST/5% milk. The peroxidase signal was developed (Supersignal West DURA kit, Life Technologies), imaged (FluorChem E, Bio‐Techne), and quantitated by densitometry (Image J).

### 2.6 Isotopic influx experiments

Unidirectional ^36^Cl^−^ influx into individual oocytes previously injected with the indicated quantities of cRNA was carried out for 30 min in 148 μl NMDG‐97 and 2 μl Na^36^Cl_2_ (~2 μCi). Influx experiments were terminated with three washes of oocytes in ice‐cold isotonic NMDG chloride solution. Washed oocytes were individually lysed in 150 μl 2% sodium dodecyl sulfate (SDS). Triplicate 10 μl aliquots of influx bath solution were used to calculate the specific activity of radiolabeled substrate ions. Oocyte ion uptakes were calculated from cpm values of cold‐washed oocytes and from bath‐specific activity.

### 2.7 Isotopic efflux experiments

For unidirectional ^36^Cl^−^ efflux studies individual oocytes previously injected with the indicated cRNA quantities were injected with 50 nL of 260 mM Na^36^Cl (20–24,000 cpm). After a 5–10 min recovery period in Cl^−^‐free ND‐96, the efflux assay was initiated by transfer of individual oocytes to 6‐ml borosilicate glass tubes, each containing 1 ml efflux solution. At intervals of 1 min over a 5 min period, 0.95 ml of this efflux solution was removed for scintillation counting and replaced with an equal volume of fresh efflux solution. After two 15 min exposure periods in Cl^−^‐free ND‐96 containing α‐ketoglutarate (1–3 mM as indicated), oocytes were placed in ND96 containing the same concentration of a‐ketoglutarate and four 1 min efflux samples were taken for counting. After completion of the assay with four final 1 min efflux samples in the presence of Cl^−^‐free ND‐96 lacking α‐ketoglutarate, each oocyte was individually lysed in 150 μl of 2% SDS. Samples were counted 3–5 min such the magnitude of 2SD was <5% of the sample mean.

### 2.8 Statistics

Data were reported as means ± SE (ion flux, ion current, fluorescence intensity). Comparisons were assessed by Student's paired or unpaired two‐tailed *t* tests (Microsoft Excel 16.16.1) or by Kruskall–Wallis ANOVA with Dunn's post‐hoc test (GraphPad Prism 9.3.1). *p* < 0.05 was interpreted as statistically significant.

## 3 RESULTS

### 3.1 OXGR1 is functionally expressed in *X* *enopus* oocytes

**FIGURE 1:**
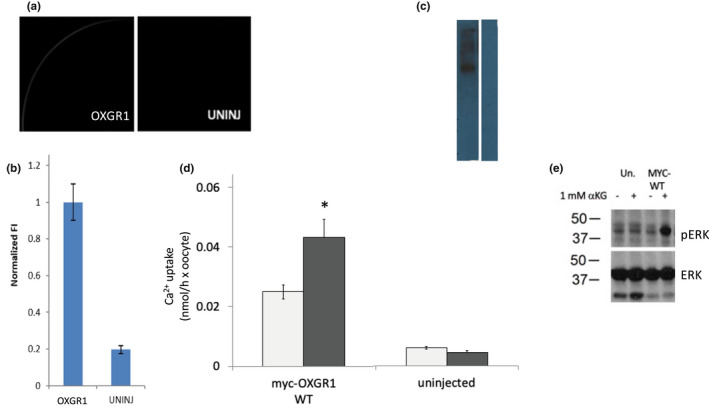
Functional expression of OXGR1 in xenopus oocytes. (a). Confocal immunolocalization of MYC‐tagged OXGR1 in a representative oocyte previously injected with 50 ng OXGR1 cRNA (left panel) and a representative uninjected xenopus oocyte (right panel). Median intensity images are shown (b). MYC immunostaining intensity at oocyte periphery in OXGR1 cRNA‐injected oocytes and in uninjected oocytes. Values are means ± s.e.m. for *n* = 11 oocytes. One of two experiments with similar results. (c). Anti‐MYC immunoblot documenting MYC‐tagged OXGR1 polypeptide expression in Xenopus oocytes (lysate from 10 oocytes). (d). Unidirectional ^45^Ca^2+^ influx into uninjected oocytes and oocytes previously injected with 10 ng MYC‐OXGR1 cRNA, in the absence (light gray bars) and presence of 1 mM αKG (dark gray bars). Values are means ± s.e.m. for *n* = 10 oocytes; *p = 0.01 by two‐way *t*‐test. (e). Representative immunoblot depicting 1 mM αKG stimulation of ERK phosphorylation in uninjected oocytes or in oocytes expressing OXGR1. One of three similar experiments.

Our ability to study Pendrin regulation in *Xenopus* oocytes first required demonstration that OXGR1 can be functionally expressed in oocytes. MYC‐tagged OXGR1 was detectable at or near the oocyte surface by confocal immunofluorescence microscopy (Figure 1a and b) and by immunoblot analysis (Figure 1c). Expression of MYC‐OXGR1 increased baseline oocyte ^45^Ca^2+^ influx, which further increased upon acute oocyte exposure to 1 mM αKG (Figure 1d). MYC‐OXGR1 expression in *Xenopus* oocytes was also accompanied by αKG‐stimulation of ERK phosphorylation (Figure 1e). These data encouraged us to test the ability of OXGR1 to stimulate Pendrin‐mediated anion exchange, as has been reported in the cortical collecting duct.

### 3.2 OXGR1 coexpression with Pendrin does not confer stimulation of Pendrin‐mediated ^36^Cl ^−^ influx by αKG

**FIGURE 2:**
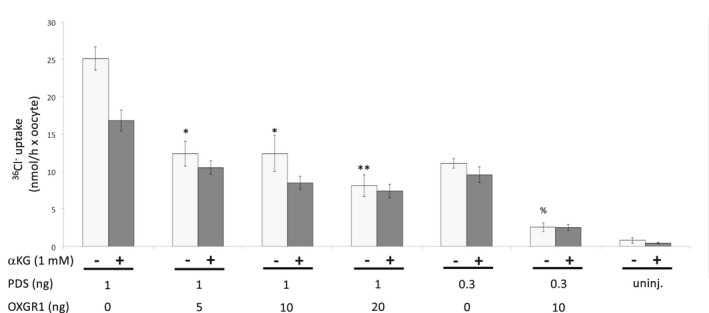
αKG (1 mM) does not stimulate Pendrin‐mediated Cl^−^ uptake into *Xenopus* oocytes. Unidirectional ^36^Cl^−^ influx at pH 7.4 into uninjected oocytes or oocytes previously injected with cRNA encoding Pendrin, with or without OXGR1 cRNA at the indicated ratios. Values are means ± s.e.m. for *n* = 10 oocytes in each condition. *p < 0.02; **p < 0.001 versus 1 ng PDS without OXGR1, both by ANOVA ^%^ *p* < 0.001 versus 0.3 ng PDS without OXGR1, by two‐tailed unpaired *t*‐test. One of two experiments with similar results.

OXGR1 and Pendrin cRNAs were co‐expressed at the ratios indicated in Figure 2. Injection of 1 ng of Pendrin cRNA alone led to robust ^36^Cl^−^ influx, at levels we have previously measured in this system (Reimold et al., 2011). 1 mM αKG failed to increase Pendrin‐mediated Cl^−^ influx activity (the apparent decrease in uptake was not statistically significant). OXGR1 co‐expression across a 4‐fold spectrum of cRNA injection ratios led to an OXGR1 cRNA‐dependent inhibition of Pendrin‐mediated ^36^Cl^−^ influx activity in the absence or presence of αKG (Figure 2). Moreover, the presence of 1 mM αKG in the bath did not stimulate Pendrin‐mediated ^36^Cl^−^ uptake at any of the Pendrin/OXGR1 cRNA ratios tested.

Since the effect of a stimulator of activity can be obscured at near‐maximal levels of that activity, the consequences of OXGR1 coexpression with Pendrin were tested at lower concentrations of injected Pendrin cRNA. Even at lower Pendrin activity, OXGR1 coexpression suppressed Pendrin‐mediated ^36^Cl^−^ uptake, and bath addition of 1 mM αKG was without effect.

### 3.3 OXGR1 coexpression with Pendrin does not confer stimulation of Pendrin‐mediated ^36^Cl ^−^ efflux by aKG

Since Pendrin‐mediated ^36^Cl^−^ influx represents Cl^−^/anion exchange, and the *Xenopus* oocyte offers the experimental advantage of the ability to modify both extracellular and intracellular conditions, we assessed the effects of OXGR1 coexpression with pendrin on ^36^Cl^−^ efflux and its sensitivity to stimulation by αKG.

**FIGURE 3:**
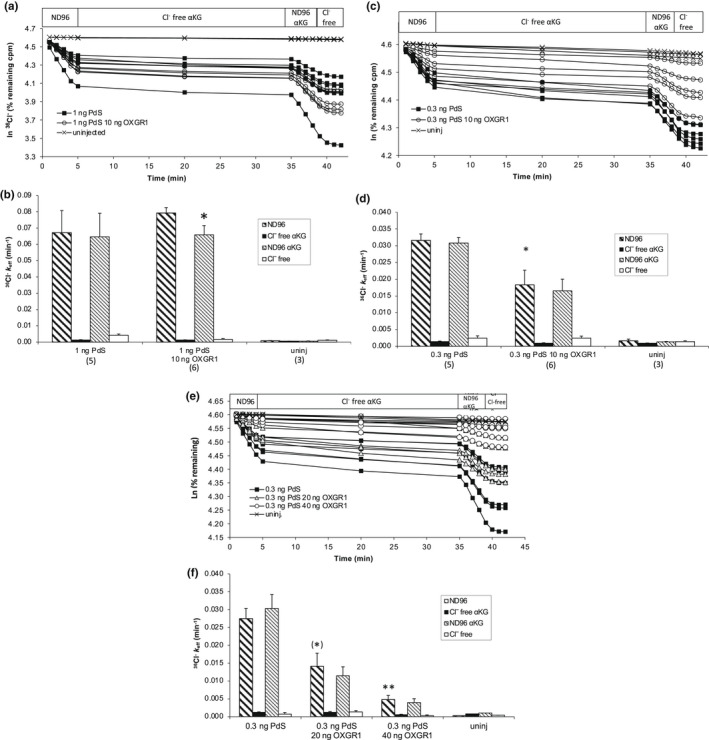
αKG (3 mM) does not stimulate Pendrin‐mediated Cl^‐^ efflux from *Xenopus* oocytes co‐injected with a range of ratios of co‐injected cRNAs encoding Pendrin and OXGR1. (a). ^36^Cl^−^ efflux traces from individual oocytes previously uninjected or coinjected with pendrin cRNA (1 ng) without or with co‐injected OXGR1 cRNA (10 ng). Oocytes were subjected sequentially to baths containing NaCl (ND96), Na cyclamate +3 mM αKG (Cl^−^‐free αKG), ND96 + 3 mM αKG, and terminating in Na cyclamate (Cl^−^‐free) as indicated. (b). ^36^Cl^−^ efflux rate constants during each efflux time period (see key, corresponding to periods of panel A) for each experimental group of oocytes as indicated for (*n*) oocytes. *p < 0.05 versus ND96, two‐tailed paired *t*‐test. (c). ^36^Cl^−^ efflux traces from individual oocytes previously uninjected or coinjected with Pendrin cRNA (0.3 ng) without or with co‐injected OXGR1 cRNA (10 ng). Oocytes were subjected sequentially to baths containing ND96, Na cyclamate +3 mM αKG, ND96 + 3 mM αKG and terminating with Na cyclamate as indicated. (d). ^36^Cl^−^ efflux rate constants during each efflux time period (see key, corresponding to periods of panel C) for each experimental group of oocytes as indicated for (*n*) oocytes. *p < 0.03 versus same condition without OXGR1; two‐tailed unpaired *t*‐test. (e). ^36^Cl^−^ efflux traces from individual oocytes previously uninjected or coinjected with Pendrin cRNA (0.3 ng) without or with co‐injected OXGR1 cRNA (20 ng or 40 ng as indicated). Oocytes were subjected sequentially to baths containing ND96, Na cyclamate +3 mM αKG, ND96 + 3 mM αKG and terminating with Na cyclamate as indicated. (f). ^36^Cl^−^ efflux rate constants during each efflux time period (see key, corresponding to periods of panel E) for each indicated experimental group of (*n*) oocytes. *p = 0.055; **p < 0.03; each versus Pds without OXGR1 in ND96, each by ANOVA.

Figure 3 demonstrates the trans‐anion dependence of Pendrin‐mediated ^36^Cl^−^ efflux from Xenopus oocytes, confirming earlier results that established Pendrin as a mediator of Cl^−^/anion (including Cl^−^/HCO_3_^−^) exchange. Figure 3a illustrates ^36^Cl^−^ efflux traces of individual oocytes first shown to express Pendrin‐mediated efflux, then exposed to aKG (3 mM) for 30 min in the presence of cyclamate bath before a brief return to ND96 in the continued presence of αKG for 4 min, followed by termination of the efflux assay in Cl^−^‐free bath in the absence of aKG to confirm maintenance of oocyte integrity. Figure 3b summarizes the ^36^Cl^−^ efflux rate constants during each experimental condition for each group of oocytes.

Prior oocyte injection of 10 ng OXGR1 cRNA together with 1 ng Pendrin cRNA failed to increase the rate of Pendrin‐mediated ^36^Cl^−^ efflux. Moreover, the coexpression of OXGR1 did not confer apparent stimulation of Pendrin‐mediated Cl^−^ efflux by bath addition of 3 mM αKG. Rather, Pendrin‐mediated ^36^Cl^−^ efflux was slightly decreased by addition of αKG (Figure 3b).

To ensure that a saturated Cl^−^ efflux rate constant did not prevent detection of Pendrin stimulation by OXGR1, oocytes previously injected with 0.3 ng Pendrin cRNA without or with coinjection of 10 ng OXGR1 cRNA were subjected to the same format of ^36^Cl^−^ efflux assay. As shown in Figure 3c and d, at this cRNA ratio Pendrin was not stimulated by 3 mM αKG; rather, Pendrin activity was suppressed by OXGR1 coexpression whether in the absence or presence of αKG.

Further increasing the injected OXGR1 cRNA to 20 or 40 ng, while maintaining Pendrin cRNA at 0.3 ng led to further proportional suppression of Pendrin activity, without conferring any stimulation of ^36^Cl^−^ efflux by 3 mM αKG (Figure 3e and f).

### 3.4 Classical protein kinase C activator, phorbol‐1‐myristate‐13‐acetate (PMA), did not significantly modulate Pendrin‐mediated ^36^Cl ^−^ efflux

**FIGURE 4:**
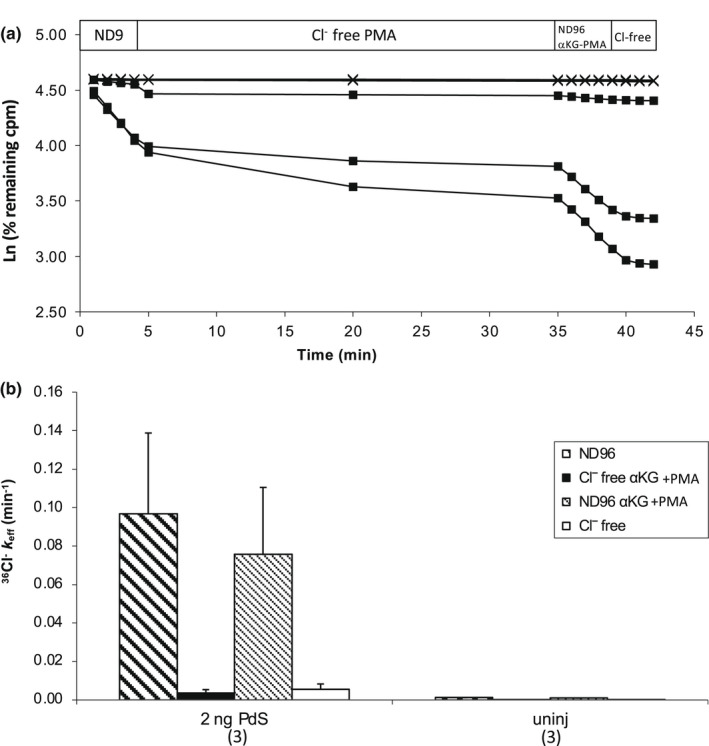
Protein kinase C activator phorbol 12‐myristate 13‐acetate (PMA) does not stimulate Pendrin‐mediated ^36^Cl^−^ efflux from *Xenopus* oocytes. (a). Representative ^36^Cl^−^ efflux traces from individual oocytes previously uninjected (upper trace with “x” time points) or injected with Pendrin cRNA (2 ng, three lower traces with black square time points), exposed sequentially to baths containing ND96, Na cyclamate containing PMA (100 nM), NaCl in the continued presence of PMA, and a terminal bath of Na cyclamate. (b). ^36^Cl^−^ efflux rate constants during each efflux time period (see key, corresponding to periods of panel A) for each indicated experimental group of (*n*) oocytes.

Pendrin stimulation by OXGR1 in the cortical collecting duct was reported to be mediated by activation of Protein Kinase C in a manner inhibited by PMA (Lazo‐Fernandez et al., 2018). However, we had earlier observed PMA‐mediated inhibition of Pendrin activity in Xenopus oocytes (Reimold et al., 2011), consistent with inhibition by PMA of the related SLC26 anion transporters SLC26A2 (Heneghan et al., 2010), SLC26A1 (Wu et al., 2016), and SLC26A6 expressed in oocytes (Hassan et al., 2007). We therefore, asked whether αKG might stimulate Pendrin in the presence of PMA. Figure 4a and b show that Pendrin was modestly (but not significantly) inhibited by PMA (100 nM) in the presence of 3 mM αKG, without evidence of stimulation.

### 3.5 aKG stimulation of OXGR1 in the presence of coexpressed Gα subunits did not activate Pendrin‐mediated Cl^−^/anion exchange

**FIGURE 5:**
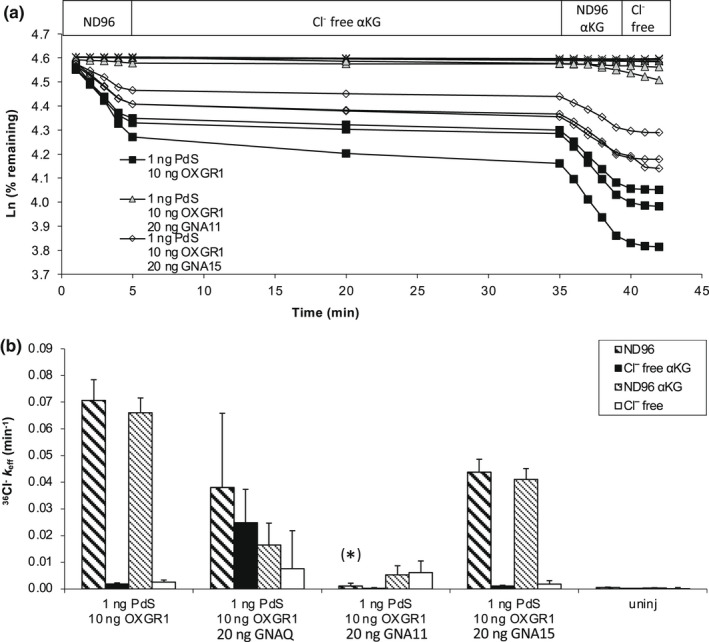
αKG does not stimulate Pendrin‐mediated ^36^Cl^−^ efflux from *Xenopus* oocytes co‐expressing Gαq, Ga11, or Gα15. (a). Representative ^36^Cl^−^ efflux traces from individual uninjected oocytes or oocytes previously injected with pendrin cRNA (1 ng) alone or with co‐injected OXGR1 cRNA (10 ng) without or with one of the coinjected cRNAs encoding Gαq, Gα11, or Gα15 (20 ng). Oocytes were subjected to sequential baths containing ND96, Na cyclamate + αKG (3 mM), ND96 + αKG (3 mM) and a terminal bath of Na cyclamate. (b). ^36^Cl^−^ efflux rate constants during each efflux time period (see key corresponding to periods of panel A) for each indicated experimental group of oocytes (*n* = 3). ^(^*^)^, *p* = 0.055 versus PDS/OXGR1 in ND96, by ANOVA across all ND96 samples.

OXGR1 signaling has long been attributed to Gαq/11 on the basis of indirect pharmacological evidence (He et al., 2004), a belief recently strengthened by results from a large‐scale chimeric G‐protein‐based signaling assay (Inoue et al., 2019). We speculated that OXGR1 coupling with Gαq/11 might be impaired in *Xenopus* oocytes. We therefore evaluated the effect of coexpression of Gαq/11 or of related Gα subunits with OXGR1 and Pendrin in *Xenopus* oocytes, to test the hypothesis that Gα protein supplementation in oocytes might unmask αKG‐stimulated Pendrin activation mediated through OXGR1 signaling. As shown in Figures 5a and b, Pendrin‐mediated ^36^Cl^−^ efflux was insensitive to αKG‐stimulation of OXGR1. Coinjection with Pendrin cRNA of cRNA encoding Gαq, Gα11, or the more broadly active Gα15, did not confer aKG‐stimulation of pendrin activity. Rather, coexpression of Gα11 attenuated (at borderline significance) Pendrin activity in the absence or presence of αKG. Moreover, coexpression of Gα15 or of Gαq partially were without significant effect on Pendrin activity and failed to confer stimulation by αKG. These experiments suggested that failure of oocytes to express αKG‐stimulated, OXGR1‐mediated increase in Pendrin's anion transport activity was not attributable to oocyte deficiency of G protein signaling in the OXGR1 pathway.

### 3.6 Tests of additional strategies to enhance OXGR1‐associated ^45^Ca^2^ ^+^ influx

We also tested the possibility that the αKG‐stimulated increment in Ca^2+^ influx might elevate intracellular [Ca^2+^] inadequately to upregulate Pendrin activity in *Xenopus* oocytes. We found that bath addition or injection of 1 mM orthovanadate to inhibit the PMCA Ca^2+^ ATPase of the plasma membrane (El‐Jouni et al., 2005) did not increase αKG‐stimulated Ca^2+^ influx (not shown). Decreasing bath pH from 7.4 to 5.0 to model maximal lumen acidification at end‐CCD did not increase αKG‐stimulated Ca^2+^ influx (not shown).

Brief exposure (10 min) of OXGR1‐expressing HEK‐293 cells to αKG at 37°C led to ligand‐induced internalization of OXGR1 in HEK‐293 cells (He et al., 2004). However, *Xenopus* oocytes at room temperature may less effectively traffic OXGR1 to the plasma membrane. To examine the possibility that endosomal OXGR1 might regulate Pendrin more potently than does plasmalemmal OXGR1 (Crilly & Puthenveedu, 2021; Sica et al., 2019), we tested the effects on OXGR1 of (1) αKG injection and (2) of injection or bath addition of the more highly cell‐permeable dimethyl‐αKG. However, neither dimethyl‐αKG nor injection of either drug proved more effective than bath αKG in activating OXGR1‐mediated Ca^2+^ influx (not shown).

Numerous GPCRs are capable of heterodimerization (Gomes et al., 2016; Moreno‐Delgado et al., 2020; Poll et al., 2021; Sleno et al., 2017; Takezako et al., 2017; Terrillon et al., 2004), and some heterodimerize preferentially (Quitterer et al., 2019; Song et al., 2021). To test the possibility that OXGR1 functions preferentially as a heterodimeric GPCR, we coexpressed OXGR1 with the type I angiotensin II receptor, AT1R. AT1R is expressed throughout the nephron on both apical and basolateral membranes. aKG has been shown to upregulate both tubular angiotensin II biosynthesis and the plasma renin receptor that colocalizes with OXGR1 (Guerrero et al., 2021). Although Ca^2+^ influx increased 8‐fold in response to bath addition of 100 nM angiotensin II, αKG‐dependent OXGR1‐stimulated Ca^2+^ influx was not increased by AT1R coexpression (not shown).

Although OXGR1 immunostaining has been reported to be limited to Type B‐intercalated cells of the CCD, mouse OXGR1 mRNA is expressed as abundantly in the connecting segment as in the CCD, and nearly as abundantly in the distal convoluted tubule (https://esbl.nhlbi.nih.gov/MRECA/Nephron/). Similar findings were evident in comprehensive transcriptome analyses of the rat distal nephron (https://esbl.nhlbi.nih.gov/helixweb/Database/NephronRNAseq/All_transcripts.html). We therefore, tested TRPV5 as a possible mediator or amplifier of αKG‐activated OXGR1‐stimulated Ca^2+^ uptake. Although TRPV5 mediated robust ^45^Ca^2^+ influx, co‐injection of 1 or 10 ng cRNA encoding TRPV5 together with 10–20 ng OXGR1 cRNA did not increase the αKG‐stimulated fraction of Ca^2+^ influx into oocytes (not shown).

### 3.7 SLC4A9 cRNA injection did not increase ^36^Cl ^−^ uptake into *Xenopus* oocytes

We next hypothesized that OXGR1 stimulation of Pendrin activity in intact collecting duct might require coordinated activation of the SLC4A9 Na^+^‐ or K^+^‐dependent Cl^−^/HCO_3_^−^ exchanger of the intercalated cell basolateral membrane (Chambrey et al., 2013; Hentschke et al., 2009; Purkerson et al., 2014), even in the relatively non‐polarized *Xenopus* oocyte. However, we found that injection into *Xenopus* oocytes of neither 50 ng human SLC4A9 cRNA nor of 50 ng mouse Slc4a9 cRNA led to detectably increased ^36^Cl^−^ uptake, even after acute oocyte loading with NaHCO_3_ (injection of estimated 50 mM final intracellular concentration) 5 min prior to initiation of the Cl^−^ influx assay (n = 10 for each condition). We similarly found no detectable SLC4A9‐mediated ^36^Cl^−^ efflux dependent upon bath NaHCO_3_ (n = 7 for each condition, data not shown). Since SLC4A9 function as a cation‐dependent Cl^−^/HCO_3_^−^ exchanger has been demonstrated in CHO‐K1 cells at 37°C (Pena‐Munzenmayer et al., 2016), we speculate that, at room temperature in the *Xenopus* oocyte, SLC4A9 fails to undergo normal trafficking, or operates at rates below our detection threshold in the oocyte. The oocyte may lack one or more proteins essential for SLC4A9 trafficking, scaffolding, or another function required for SLC4A9 expression, or the orthologous frog proteins may not interact well with the human transporter.

## 4 DISCUSSION

We have explored the utility of the *Xenopus* oocyte as an experimental system in which to further understand the mechanism by which the αKG receptor, OXGR1 activates Pendrin‐mediated Cl^−^/HCO_3_^−^ exchange in the Type B and non‐A non‐B intercalated cells of the cortical collecting duct. We found that OXGR1 was functionally expressed in the oocyte as judged by αKG‐stimulated upregulation of ^45^Ca^2+^ influx and ERK phosphorylation. However, αKG‐stimulated OXGR1 failed to activate the Cl^−^/HCO_3_^−^ exchange activity of co‐expressed human Pendrin in *Xenopus* oocytes, whether measured as Cl^−^ influx or as trans‐anion‐dependent Cl^−^ efflux. This lack of activation was evident at high or at low levels of Pendrin expression, and at OXGR1/Pendrin cRNA mass ratios between 5 and 30. At every tested level of Pendrin expression, coexpression of OXGR1 led to suppression of Pendrin activity whether in the absence or presence of αKG. Unlike in mouse CCD, Pendrin activity in the *Xenopus* oocyte was not stimulated by the PKCα/δ activator PMA. Moreover, coexpression with Pendrin and OXGR1 of the Gα subunits Gαq, Gα11, or Gα15 either inhibited or were of no significant effect on pendrin activity. Heterologous expression of SLC4A9 activity in the *Xenopus* oocyte was similarly below our threshold of detection, preventing test of the hypothesis that OXGR1 might primarily regulate the basolateral cation‐dependent Cl^−^/HCO_3_^−^ exchanger SLC4A9 in CCD Type B‐IC. Coexpression of TRPV5 in Xenopus oocytes also failed to elicit OXGR1 stimulation of Pendrin activity.

The above data suggest possible interference by OXGR1 of Pendrin trafficking from the endoplasmic reticulum to plasma membrane, perhaps through competition for a concentration‐limiting chaperonin or other cofactor required for protein folding and/or trafficking. The latter might include the recently reported pendrin‐interactor and modulator, IQGAP1 (Xu et al., 2022). As the conserved domains of *X. laevis* and human IQGAP1 exhibit from 67%–72% (for IQGAP repeats and IQ motifs) up to 93%–97% amino acid sequence identity (RasGAP‐related and calponin homology domains) (Yamashiro et al., 2003), with the overall identity of 78%, the ability of *X. laevis* IQGAP1 to regulate human Pendrin is certainly plausible. However, *X. laevis* IQGAP1 is a maternal transcript of the oocyte, and IQGAP1 polypeptide is expressed in egg and later stage embryonic tissues.

Alternatively, one or more components involved in the biosynthesis, folding, and trafficking of OXGR1, Pendrin, and other components of the signaling pathways linking the two in Type B‐IC may function inefficiently at the physiological room temperature of amphibians. Yet to be addressed is the possibility that either coexpression of the Type B‐IC apical membrane Na^+^‐dependent Cl^−^/HCO_3_^−^ exchanger, SLC4A8 (Leviel et al., 2010) or coexpression of the Type B‐IC apical membrane K‐Cl cotransporter, KCC3a (Ferdaus et al., 2022) (or both) may be required for OXGR regulation of pendrin. (Note, however, that SLC4A8 localization has more recently been reported in the basolateral rather than the apical membrane of Type B‐IC (Xu et al., 2018)).

The 1–3 mM extracellular αKG concentrations chosen for our experiments may have led to desensitization of oocyte‐expressed OXGR1 (αKG EC50 30–70 nM in HEK‐293 cells (He et al., 2004)), such that OXGR1 may have become unresponsive to extracellular αKG during the 5 min preincubation and the ~30 min flux assays. Such GPCR desensitization is compatible with documented OXGR1 recruitment of endogenous oocyte β‐arrestin (Southern et al., 2013), although β‐arrestin could also mediate stimulatory limbs of OXGR1 signaling (Ahn et al., 2020). However, urinary [αKG] can be as high as 2–3 mM, and can rise to 36 mM after alkali loading (Tokonami et al., 2013), suggesting that our αKG concentrations did not exceed physiological limits. The suppression of Pendrin activity seen with coexpression of OXGR1 and Gα subunits might also reflect a need for molar co‐expression in oocytes of specific isoforms of Gβγ subunits. This suppression of Pendrin activity might be further explored by coexpression with the constitutively active Gαq mutant R183Q that underlies vascular malformations in 80% of patients with Sturge–Weber Syndrome (Shirley et al., 2013).

GPCR are usually homodimeric, but several GPCR have been shown also to function as heterodimers, often with distinct patterns of signaling (Gomes et al., 2016). As the AT1R angiotensin II receptor is widely expressed throughout the nephron in both apical and basolateral membranes, we tested the hypothesis that OXGR1 might heterodimerize or exhibit cross‐talk with AT1R. However, we detected no evidence of αKG‐stimulated Pendrin activity resulting from coexpression of OXGR1 with AT1R in *Xenopus* oocytes.

Single cell RNAseq (Chen et al., 2017; Poll et al., 2021) has revealed a spectrum of GPCRs expressed at (relatively) elevated levels in Type B‐IC of mouse CCD (as opposed to Type A‐IC and Principal cells) including the Ca^2+^‐sensing receptor (*Casr*), the β2‐adrenergic receptor (*Adrb2*) and the type 3 prostaglandin E receptor (*Ptger3*). Also expressed at levels similar to or below those in Type A‐IC are the glucagon receptor (*Gcgr*), the Vasopressin 1A receptor (Avpr1a), the parathyroid hormone receptor (*Pth1r*), and the thyroid‐stimulating hormone receptor (*Tshr*). Any of these gene products might be considered candidates for heterodimerization with OXGR1 in Type B‐IC. (Note, however, that in contrast to the reported selective immunolocalization of OXGR1 in Type B‐IC, OXGR1 mRNA as measured by scRNAseq was found to be twice as abundant in Type A‐IC as in Type B‐IC (Chen et al., 2017)).

OXGR1 is expressed in mast cells, where it is induced by engagement of the IgE receptor, FcεR1. αKG at high concentrations can reduce IgE‐triggered histamine release (Nishi et al., 2016). A recent genetic screen for modulators of histamine receptor H1R‐stimulated Ca^2+^_i_ signaling in HeLa cells revealed OXGR1 knockdown as a strong inhibitor of H1R signaling, but heterologous OXGR1 overexpression failed to rescue this anti‐histaminergic phenotype. Interpretation of this result, however, was complicated by reduced efficiency of complex glycosylation of OXGR1, suggesting abnormal OXGR1 trafficking. The histamine receptors *HTHR1‐4* are indeed expressed in rodent cortical distal nephron, albeit at low levels (https://esbl.nhlbi.nih.gov/MRECA/Nephron/) (Chen et al., 2017).

Our failure to observe Pendrin stimulation by PMA in *Xenopus* oocytes may reflect tissue‐specificity of PKC isoform expression or activity, as the Pendrin‐related SLC26 anion exchangers SLC26A2 (Heneghan et al., 2010), SLC26A3, and SLC26A6 (Stewart et al., 2011) were inhibited by PMA in *Xenopus* oocytes. Moreover, pancreatic ductal bicarbonate secretion (likely mediated in substantial part by SLC26A3 and SLC26A6, as well as by CFTR) was also inhibited by PMA (Hegyi et al., 2006). Substance P is the hormonal inhibitor of pancreatic bicarbonate secretion that (directly or indirectly) inhibits protein kinase C in pancreatic duct (Hegyi et al., 2005). However, Substance P released from renal sensory nerves by prostaglandin E2 (Kopp et al., 2002) also acts on the Type B‐IC of the cortical collecting duct via adenylyl cyclase to activate bicarbonate secretion dependent upon both CFTR and Pendrin (Berg et al., 2021). A connection between Substance P and protein kinase C stimulation of Pendrin activity has not been reported.

OXGR1 is a receptor not only for αKG (He et al., 2004) but also for cysteinyl leukotriene E4, with an EC50 of 2.5 nM (Kanaoka et al., 2013). Leukotriene E4 is synthesized and metabolized in the kidney (Abraham et al., 1995), and urinary LTE4 declines in parallel with declining eGFR (Rafnsson & Back, 2013). It remains possible that LTE4 may play a role in OXGR1 regulation of Pendrin, but tests of this hypothesis remain unreported.

OXGR1 is also one of the multiple GPCRs that can serve as a cellular entry pathway for several strains of influenza virus (Orr‐Burks et al., 2021), and is essential for optimal viral proliferation. OXGR1 is expressed in Glp‐1‐positive enteroendocrine cells (Roberts et al., 2019) and has been variously been implicated in respiratory epithelial mucin production (Bankova et al., 2016), hypertrophic cardiomyopathy (Omede et al., 2016), fibroblast proliferation (Wang et al., 2019), and axon growth but not guidance (Cherif et al., 2018). An OXGR1 variant of unknown significance has also been linked to early ischemic stroke in families with a history of stroke (Ilinca et al., 2020). Thus, future research on OXGR1 signaling and its possible integration with additional non‐OXGR1‐dependent metabolic effects of extracellular αKG should be of wide interest within as well as beyond nephrology.

## Disclosures

S.L. Alper and L. Michael Snyder are Consultants to Quest Diagnostics.

J. G. Wohlgemuth and J. S. Dlott are employees and stockholders of Quest Diagnostics.

F.Hildebrandt was a co‐founder of Goldfinch Biopharma Inc.

The other authors declare that they have no competing financial interests. No part of this manuscript has been previously published.
